# Shorebird loss increases soil CO_2_ emissions in coastal wetlands under restoration

**DOI:** 10.1016/j.fmre.2025.08.015

**Published:** 2025-09-15

**Authors:** Chunming Li, Yizhou Sun, Peter Müller, Mark D. Bertness, Baoshan Cui, Lijuan Cui, Bo Li, Qiang He

**Affiliations:** a State Key Laboratory of Wetland Conservation and Restoration, National Observation and Research Station for Wetland Ecosystems of the Yangtze Estuary, Ministry of Education Key Laboratory for Biodiversity Science and Ecological Engineering, School of Life Sciences, Fudan University, Shanghai 200438, China; b Institute for Environmental Sciences, University of Kaiserslautern-Landau, 76829 Landau, Germany; c Department of Ecology, Evolution and Organismal Biology, Brown University, Providence, RI 02912, USA; d State Key Laboratory of Wetland Conservation and Restoration, School of Environment, Beijing Normal University, Beijing 100875, China; e State Key Laboratory of Wetland Conservation and Restoration, Institute of Wetland Research, Chinese Academy of Forestry, Beijing 100091, China

**Keywords:** Blue carbon, Keystone species, Shorebirds, Top-down control, Nature-based solutions, Soil respiration, Bioturbation

## Abstract

Countries worldwide are increasingly adopting nature-based solutions (NbS) to tackle the challenges of global climate change. One recently proposed NbS is wildlife conservation, yet the impact of wildlife on carbon emissions in ecosystems under restoration remains poorly understood. We assessed the role of shorebirds in regulating the carbon cycle of a coastal wetland under vegetation restoration, using a long-term field experiment that simulated the exacerbation of ongoing shorebird loss through shorebird exclusion. We found that compared to control treatments, shorebird exclusion significantly increased soil CO_2_ emissions by 105% on average over four years, an impact that was particularly pronounced in seasons (spring and autumn) when migratory shorebirds were abundant. The impact of shorebird exclusion on CO_2_ emissions was mainly driven by the release of crab bioturbators from predator control, with CO_2_ emissions increasing strongly as crab bioturbator abundance increased. Largely owing to increased soil CO_2_ emissions, shorebird exclusion significantly increased ecosystem respiration and net ecosystem exchange and reduced soil carbon sequestration, although it did not affect gross ecosystem photosynthesis, litter decomposition, microbial biomass carbon, or dissolved organic carbon. Our findings underscore the substantial, but previously overlooked role shorebirds play in regulating the carbon cycle of coastal wetlands under vegetation restoration. Conservation of shorebirds and other predators of soil bioturbators may be a valuable NbS to help curb global warming.

## Graphical abstract

**Figure:**
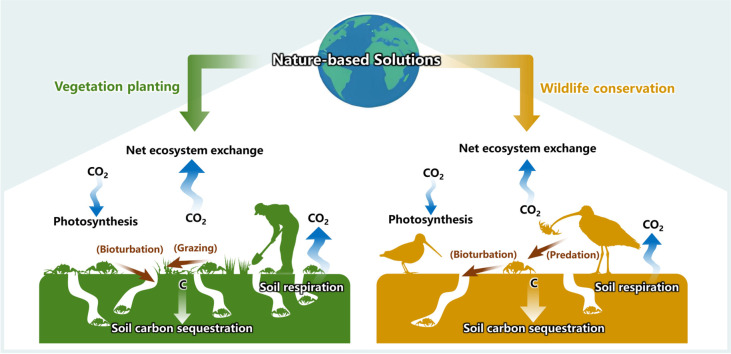

## 1 Introduction

Countries worldwide are increasingly committed to adopting Nature-based Solutions (NbS) to capture atmospheric CO_2_ and store it in terrestrial and marine ecosystems [1,2]. NbSs were first mentioned in 2008 by the World Bank, and refer to the protection, sustainable management, and restoration of natural or modified ecosystems that address societal challenges effectively and adaptively, simultaneously providing human wellbeing and biodiversity benefits [3,4]. To address the challenges of rapid climate change, NbSs have been proposed as cost-effective and sustainable strategies for reducing carbon emissions [5,6]. NbSs aim to avoid carbon emissions of 4 GtCO_2_ each year by protecting ecosystems, including coastal wetlands, and to capture and store 5–6 GtCO_2_ more each year by restoring and managing plants, soils and sediments in these ecosystems [[6], [7], [8]]. However, how to achieve these targets remains largely uncertain.

As a key approach of NbS, restoration has typically focused on reestablishing vegetation or other foundation species such as oysters at basal trophic levels. Such restoration projects posit that vegetation or other foundation species determine the capacity of ecosystems to capture and store carbon [8,9]. However, even decades following the implementations of such restoration projects, the recovery of degraded ecosystems and their ability to store carbon often remains slow or incomplete [[10], [11], [12]], in part owing to the strong grazing pressure of herbivores [13]. In fact, it is increasingly acknowledged that the capacity of ecosystems for carbon capture and storage is not solely contingent upon plants, but also profoundly governed by animals, including herbivores and predators at upper trophic levels [[14], [15], [16]]. Predator loss can feed back to lower trophic levels like plants through trophic cascades, thereby dramatically altering their capacity to fix and store carbon [[17], [18], [19]]. While wildlife conservation, which includes many predators, is increasingly discussed as a pivotal NbS [8,19,20], how predators affect the carbon cycle of ecosystems under restoration still lacks experimental evidence.

In this study, we assessed how shorebirds–key wetland predators currently facing widespread declines–affected the carbon cycle of coastal wetlands under restoration. Coastal wetlands worldwide cover only 0.2% of the ocean surface but contribute to roughly 50% of the ocean carbon burial [21,22]. In the Yellow Sea where our study is focused, coastal wetlands are not only critical for carbon sequestration [23,24], but also provide important stopover habitats for numerous shorebird species migrating along the East Asian-Australasian Flyway [[25], [26], [27]]. These wetlands, however, have been severely degraded by anthropogenic impacts, including the invasion of the exotic smooth cordgrass *Spartina alterniflora* since the mid-1990s, contributing to a > 80% decline in shorebird abundance [16,28]. Shorebird loss has been shown to release their prey (such as burrowing crabs that are the primary bioturbators in these wetlands) from predator control and trigger outbreaks of crab bioturbators [16]. Although crab bioturbation has been shown to increase soil respiration in coastal wetlands and reduce their potential for carbon sequestration [29], the impact of lost top-down control by shorebirds on the carbon cycle of these ecosystems remains untested.

**Fig. 1:**
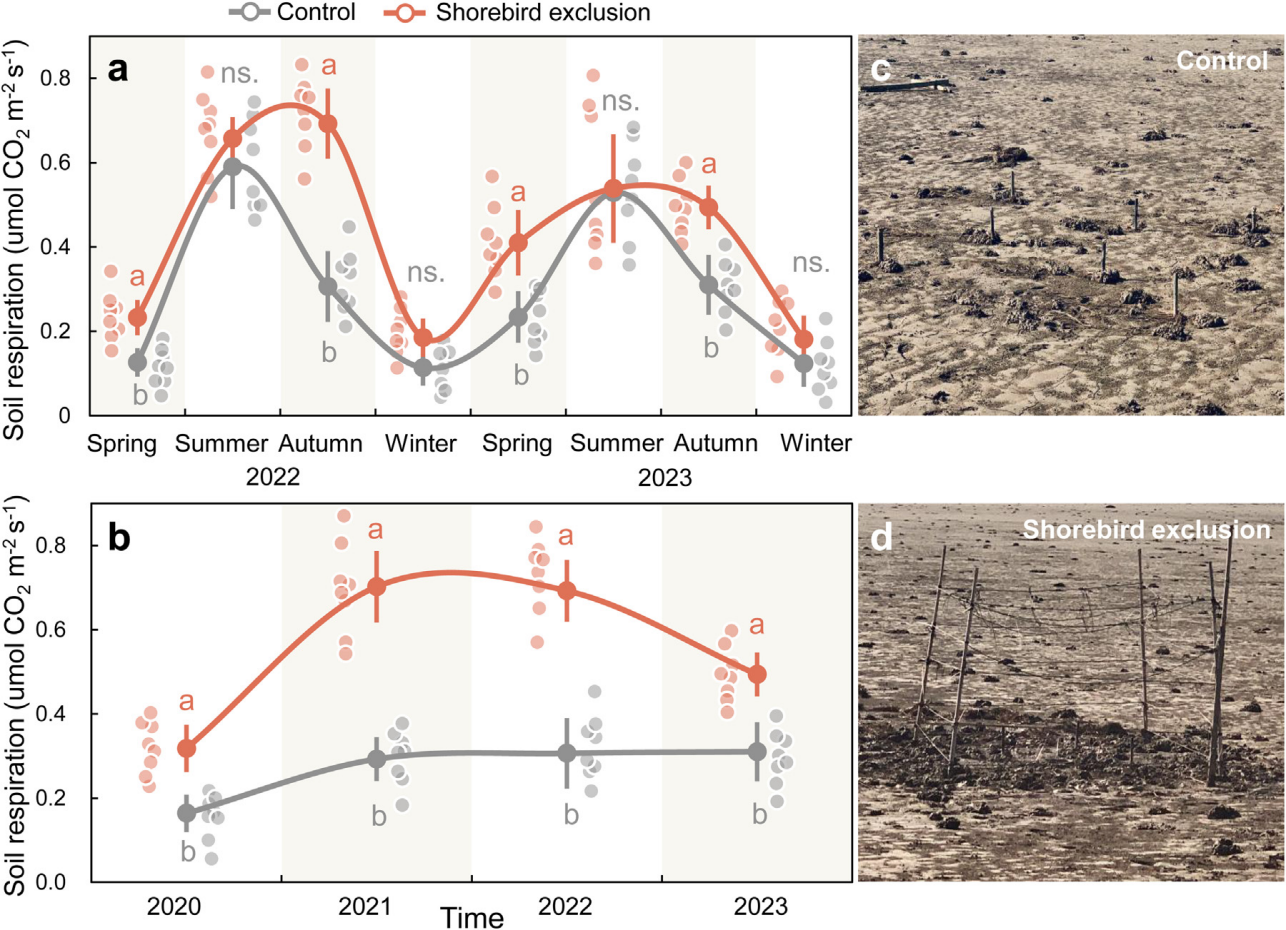
**Soil respiration in control and shorebird exclusion treatments.** (a) Seasonal variation in soil respiration from 2022 to 2023. (b) Interannual variation in soil respiration in autumn of 2020–2022. Data are shown as means ± SE (*n* = 8). (c) Photograph showing a representative control plot. (d) Photograph showing a representative shorebird exclusion treatment. Treatments within a period that do not share a letter (i.e., a and b) differ significantly from one another (*P* < 0.05; *P*-values calculated using the *t*-test indicate statistical significance between the two treatments).

We hypothesized that shorebirds play a crucial role in mediating carbon cycling in these coastal wetlands by controlling crab bioturbator populations. To test this hypothesis, we initiated a shorebird exclusion experiment in 2018 in a coastal wetland under restoration in the Yangtze Estuary, where exotic cordgrass has been successfully eradicated since 2015, but native vegetation has not yet recovered. We focused on two treatments in the field experiment (Fig. 1c-d): i) control: plots that represented shorebird populations in the current natural state; and ii) shorebird exclusion: shorebirds were experimentally excluded to simulate exacerbated shorebird population loss. Then, to assess the impact of shorebird loss on soil CO_2_ emissions, we quantified soil respiration in different experimental treatments from 2020 to 2023. To tease apart the effects of shorebirds versus abiotic factors (e.g., soil temperature) on soil respiration, we further constructed a structural equation model (SEM). Finally, in addition to soil respiration, we investigated the impacts of shorebird loss on multiple other processes of the carbon cycle, such as net ecosystem exchange, gross ecosystem photosynthesis and soil carbon sequestration.

## 2 Methods

### 2.1 Study site

Our field experiment was conducted in a coastal wetland in Shanghai Chongming Dongtan Bird National Nature Reserve, a World Natural Heritage and Wetland of International Importance site, located in the Yangtze Estuary, China (31°25′ N–31°38′ N, 121°50′ E–122°05′ E). The climate is subtropical monsoonal, with an average annual rainfall of 1022 mm and a mean annual temperature of 15.3 °C. This site has irregular semidiurnal tides with a mean tidal range of 2.0–3.1 m [30]. Soil pore water salinity ranges from 4 to 18 ppt [31,32].

The study site is a vast estuarine tidal wetland that was once largely dominated by *Scirpus mariqueter*. In the mid-1990s, the introduction of exotic cordgrass led to dramatic changes in these coastal wetlands. Over the past three decades, exotic cordgrass spread rapidly and replaced large areas of *Scirpus*, which is currently highly threatened in the Yangtze Estuary [16]. In 2015, a large-scale cordgrass eradication project was carried out, which successfully removed the exotic cordgrass [32]. However, even ten years after eradication, the area remains largely a bare mudflat, with little recovery of native vegetation including *Scirpus*.

The study site serves as a crucial stopover for migratory shorebirds along the East Asian-Australasian Flyway [33]. The most abundant shorebirds at the restoration site were Kentish plovers (*Charadrius alexandrinus*), Dunlin (*Calidris alpina*), and Great knot (*Calidris tenuirostris*) [16]. The most conspicuous macrofauna were burrowing crabs, particularly *Sesarma dehaani* and *Helice tientsinensis*, which are key bioturbators in these coastal wetlands [16,32]. Shorebirds, as top predators in these coastal wetlands, feed on macrofauna, including burrowing crabs [16]. Our previous studies have shown that shorebird exclusion led to periodic outbreaks of burrowing crab populations in spring and autumn, while having negligible impacts on other macrofauna [16,34].

### 2.2 Experimental design

In October 2018, we established the Coastal Wetland Trophic Restoration Experiment in the *Spartina* eradication area (see Li et al. for detailed experimental design [16]). In this study, we focused on two treatments that were explicit for testing the effects of shorebirds: control and shorebird exclusion treatments. Both treatments were replicated eight times in 2 m × 2 m plots marked with wooden corner stakes (Fig. 1c-d). All plots had similar elevations (< 5 cm difference) and crab burrow densities (8–10 burrows per plot). All plots had no pre-existing vegetation and were initially planted with nine sods of *Scirpus* (each containing 15 ramets of similar size, 15–20 cm tall), arranged in a 3 × 3 grid with even spacing. Planting was conducted in May 2019, and the *Scirpus* was excavated from a nearby vegetated area. All planted *Scirpus* were eliminated by crab grazers in a couple of weeks, leaving no plants in both treatments.

Control plots received no additional treatments (Fig. 1c). Shorebird exclusion plots were established by attaching five lines of dark green nylon strings to wooden corner stakes to prevent shorebirds from entering the plots (Fig. 1d). Strings at the bottom and top were positioned approximately 5 cm and 120 cm above the marsh surface, respectively, while the remaining strings were spaced with roughly 10–30 cm intervals. The same nylon strings (arranged in a 3 × 3 pattern, with even spacing) were used to cover the top of the plots to prevent birds from flying into the plots. This design successfully minimized shorebird intrusion, achieving a reduction in shorebird footprints of >80% [16,34], while allowing other predators such as fish and green crabs to traverse the plots freely. Similar bird exclusion methods have also been successfully used previously [16,34,35]. We conducted regular monitoring every 1–2 weeks, and treatments were maintained as necessary.

### 2.3 Effects of shorebirds on soil CO_2_ emissions

To examine the impact of shorebird exclusion treatments on CO_2_ emissions from soils, we quantified soil respiration in each plot in four seasons (January, April, July, and October) from 2022 to 2023. Soil respiration is widely recognized as the primary source of soil carbon efflux (i.e., soil CO_2_ emissions) to the atmosphere [36,37]. To measure soil respiration, we first installed a PVC collar (20 cm diameter, 20 cm depth) in each plot. Following previous studies on coastal wetlands [29,38,39], we measured soil respiration between 09:00 and 11:30 AM at low tides during neap tide periods on sunny days, using a portable LI-8100A soil CO_2_ flux system (LI-COR, Lincoln, Nebraska, USA). During neap tides, soil moisture and gas exchange conditions were relatively stable, and the wetland surface was exposed for a longer period, making field measurements of soil respiration feasible. Differences in soil respiration (μmol CO_2_ m^−2^ s^−1^) between experimental treatments were analyzed using a linear mixed-effects model, with treatment and season as the fixed effects and plot ID as the random effect. We then compared soil respiration between experimental treatments in each season using the independent two-sample *t*-test. All data were tested for normality using the Kolmogorov-Smirnov test. Statistical results were considered significant at the level of *P* < 0.05 throughout this study.

To assess if the impacts of shorebird exclusion were consistent among different years, we further measured soil respiration in the two treatments in each year from 2020 to 2023, all in October when migratory shorebirds were abundant. As described above, all measurements were taken between 9:00 and 11:30 AM at low tides during neap tide periods on sunny days. We compared soil respiration between experimental treatments in each year using the independent two-sample *t*-test. Differences in soil respiration between experimental treatments were analyzed by using a linear mixed-effects model, with treatment and year as the fixed effects and plot ID as the random effect. All data were tested for normality using the Kolmogorov-Smirnov test. Statistical results were considered significant at the level of *P* < 0.05 throughout this study.

### 2.4 Effects of shorebirds versus other factors

To investigate the effects of shorebirds in comparison to other biotic and abiotic factors, we quantified several other factors, including crab abundance, soil temperature, soil moisture, soil pore water salinity, and soil pH in different treatments during each soil respiration measurement. To quantify crab abundance in each treatment, we counted the number of crab burrows in the center (1 m^2^) of each plot, which served as an indicator of crab abundance and bioturbation, following previous studies [40,41].

Soil temperature was recorded at 15 cm depth near each PVC collar using a Delta-T soil thermometer. To quantify soil moisture, soil pore water salinity, and soil pH, three soil cores (2.5 cm diameter, 15 cm depth) were collected from each plot, mixed into a composite sample, and transported to the laboratory in ziplock bags for analysis. Fresh soil samples were weighed, oven-dried at 60 °C for 48 h, and reweighed to determine soil moisture (%). To determine soil pore water salinity and pH, 16 g of dried soil was mixed with 80 mL of deionized water, shaken on an oscillator for 1 min, and the pH and salinity of the supernatant were measured after standing for 30 min. Soil pH and salinity were measured using a pH meter (6010M, Jenco Electronics, USA) and a portable conductivity meter (EC3840, Jenco Electronics, USA), respectively. Soil pore water salinity was calculated based on the salinity of the supernatant and the initial moisture content of the soil core [42].

To assess the effects of these biotic and abiotic factors on soil respiration, we first conducted linear and nonlinear quadratic regressions using the data in each plot in four seasons (January, April, July, and October) from 2022 to 2023. We selected the best-fitting linear or nonlinear model based on the Akaike information criterion corrected for small sample sizes (AICc). To examine the potential interactive effects of temperature and shorebird exclusion on soil respiration, we quantified the effect size of shorebird exclusion on soil respiration as the natural log of shorebird exclusion treatments divided by control treatments. Then we constructed linear and quadratic models to examine how the shorebird effect size on soil respiration changed with soil temperature, and selected the better-fitting model based on AICc.

We further constructed an SEM to tease apart the effects of shorebird exclusion, crab abundance, soil temperature, soil moisture, soil pH, and soil pore water salinity on soil respiration [43]. The SEM was based on three hypotheses: (1) crab abundance, soil temperature, soil moisture, soil pH, and soil pore water salinity can affect soil respiration [29,[44], [45], [46], [47]]. (2) Shorebird exclusion, soil temperature, soil moisture, soil pH, and soil pore water salinity can affect crab abundance [16,48]. (3) Soil temperature and pore water salinity can affect soil moisture and soil pH, respectively [49,50]. Data were standardized using z-scores. All data were tested for normality using the Kolmogorov-Smirnov test, and variables were log-transformed where necessary (i.e., crab abundance). Model fit was evaluated using Fisher’s exact test and *P*-value, and a small Fisher’s C statistic value and *P*-value greater than 0.05 indicated a good fit of the model results. All analyses were performed using R version 4.0.3 with the ‘lavaan’ package for SEM analyses.

### 2.5 Effects of shorebirds on the carbon cycle

To evaluate the effects of shorebird exclusion on additional measures of the carbon cycle besides soil respiration, we quantified ecosystem respiration, gross ecosystem photosynthesis, net ecosystem exchange, soil dissolved organic carbon, soil microbial biomass carbon, litter decomposition, and soil carbon sequestration in each plot in 2023. We quantified ecosystem respiration and net ecosystem exchange on sunny days between 09:00 and 11:30 AM during neap tide in October using a portable LI-8100A soil CO_2_ flux system with transparent and lightproof chambers. We then calculated gross ecosystem photosynthesis as the difference between ecosystem respiration and net ecosystem exchange in each plot.

To quantify soil dissolved organic carbon and microbial biomass carbon, we followed standard methods [51]. Specifically, in October 2023, three soil cores (2.5 cm diameter, 15 cm depth) were collected from each plot, mixed into a composite sample, and transported to the laboratory in ziplock bags. Then, 1 *g* of soil was weighed, added to 40 mL centrifuge tubes, and assigned to either a fumigated or non-fumigated treatment. Fumigated samples were exposed to gaseous chloroform for 24 h in a glass desiccator. After 24 h, the samples were extracted with 25 mL of 0.5 M K_2_SO_4_, shaken in an orbital shaker for 1 h at 25 °C and 150 rpm. Following incubation, the samples were centrifuged for 10 min at 10 °C and 5000 rpm. The supernatant was vacuum filtered through 0.45 μm filters, acidified with double-distilled H_2_SO_4_ for preservation, and stored at 4 °C until analysis. To quantify soil dissolved organic carbon, non-fumigated samples were processed in the same manner but without chloroform fumigation. Soil dissolved organic carbon was determined with a TOC Analyzer. Microbial biomass carbon was calculated as the difference between the fumigated and the non-fumigated samples.

To quantify litter decomposition rate, standing dead *Scirpus* stems were collected from a vegetated area, washed, and desiccated at 65 °C until they reached constant weight. Ten grams of these prepared stems were distributed into each of 48 mesh bags (15 cm wide × 20 cm long, 1 mm mesh size). Three mesh bags were placed on the sediment surface in each plot in August. Following intervals of 1, 2, and 3 months, one of the three mesh bags in each plot was retrieved, and the remaining material was washed, dried, and weighed. An exponential equation fitting *Scirpus* mass remaining against time was then constructed: *L_t_* = *L_0_* e^–^*^kt^*, where *L_0_* and *L_t_* are litter mass at time *0* and *t* (mo), respectively, and *k* is the decomposition rate (mo^−1^).

To quantify soil carbon sequestration, soil samples (the same as collected for determining soil dissolved organic carbon) were air-dried and sieved through a 0.1-mm mesh. We acidified 10 g of air-dried soil samples using 1 mol *L*^−1^ HCl to remove carbonates. The acidified samples were then oven-dried, ground, and sieved through a 0.1-mm mesh, and their carbon concentration (soil organic carbon %) was determined using an element analyzer (vario MACRO cube, Elementar, Germany). To quantify soil bulk density, an additional intact soil core (100 cm^3^) was collected from each plot. These cores were dried and weighed in the laboratory. Bulk density (g cm^−3^) was calculated using the equation *M*_d_ / *V*, where *M*_d_ is the mass of dry soil and *V* is the volume of the soil core. To quantify sediment accretion rate, we inserted three 1.5-m-long PVC poles vertically into the sediment at three randomly selected locations in each plot until resistance. Each pole was marked 20 cm above the substrate surface, and the distance between the mark and the substrate was re-measured monthly from April to October 2023. The sediment accretion rate was calculated as the difference between the first and last measurements divided by the number of months (cm month⁻¹). Soil carbon sequestration rate (g C m^−2^ mo^−1^) was estimated by multiplying sediment accretion rate by carbon density, where carbon density was calculated by multiplying soil organic carbon concentration (%) by soil bulk density.

To test for the effects of shorebird exclusion, we first compared these carbon cycle metrics between experimental treatments using the independent two-sample *t*-test. Spearman correlations were used to generate a bivariate correlation metric covering all eight carbon cycle metrics (including soil respiration). We further performed a principal component analysis (PCA) of the eight carbon cycle metrics in control and shorebird exclusion treatments with the function “PCA” of the R package “psych” and scores normalized by setting scale = TRUE. The first two principal components explained in total 83.2% of the variance in our data and were used to visualize differences across the eight carbon cycle metrics between the two treatments. We compared these scores from the first two principal components between experimental treatments using the Welch two-sample *t*-test. Furthermore, we calculated the mean percent difference of each carbon cycle metric. All data were tested for normality using the Kolmogorov-Smirnov test.

## 3 Results

### 3.1 Effects of shorebirds on soil CO_2_ emissions

Shorebird exclusion significantly increased wetland soil CO_2_ emissions. This effect of shorebird exclusion varied significantly with season (interaction between treatment and season, *P* = 0.0005; Table S1). Compared to control treatments, shorebird exclusion did not significantly affect soil respiration in summer and winter, but increased it by 59%−126% in spring and autumn when migratory shorebirds were abundant at the study site (Fig. 1a). The effect of shorebird exclusion was consistently significant in the four years from 2020 to 2023 (Fig. 1b), although it varied significantly with year (interaction between treatment and year, *P* = 0.0292; Table S2), being strongest in 2021 and weakest in 2020. On average, shorebird exclusion significantly increased soil respiration by 105% compared to control treatments (Fig. 1b).

### 3.2 Effects of shorebirds versus other factors

**Fig. 2:**
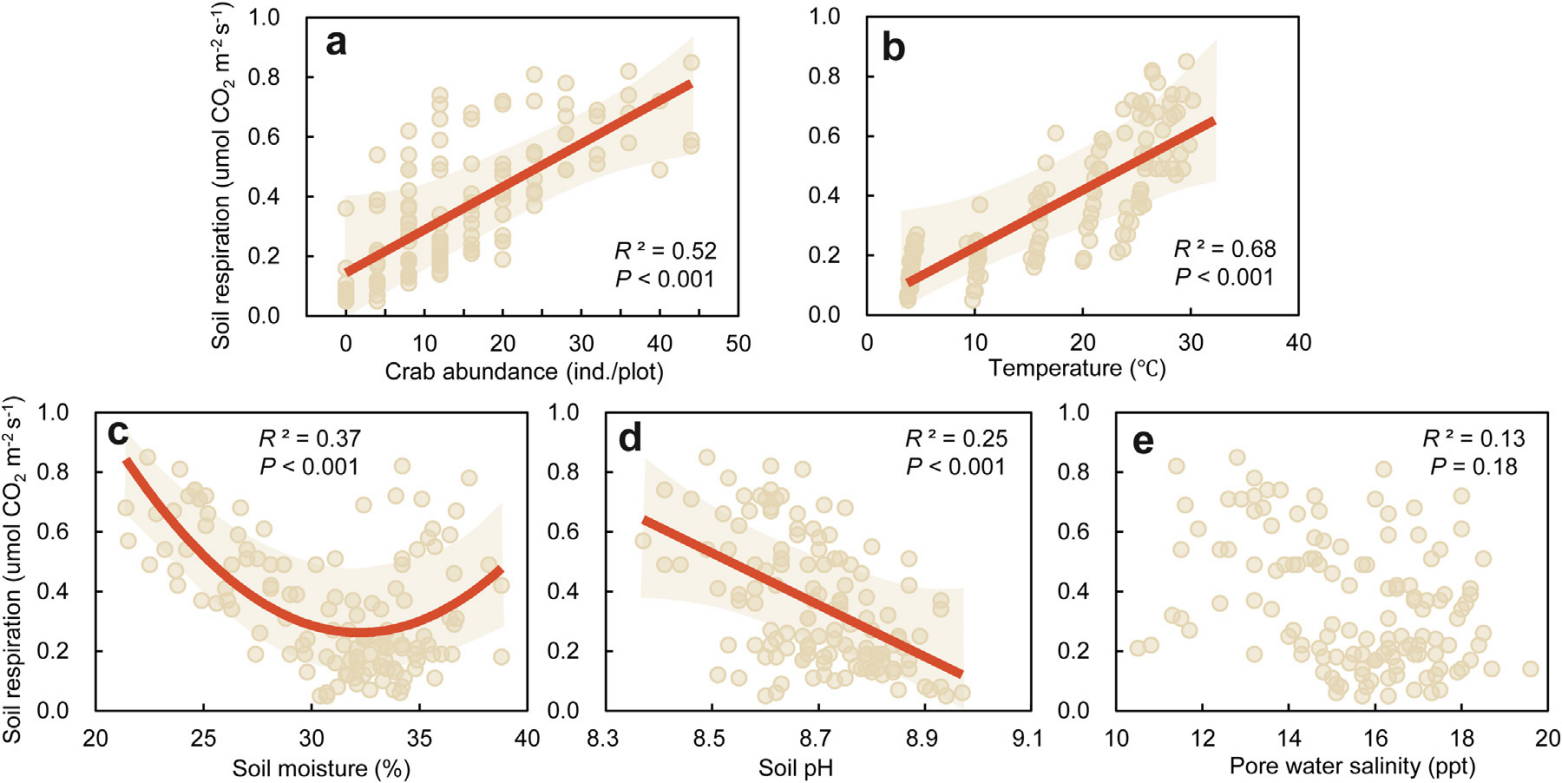
**Soil respiration as a function of crab abundance and abiotic factors.** Red lines indicate a statistically significant linear or quadratic regressions (*P* < 0.05). Shaded areas are 95% confidence intervals.

**Fig. 3:**
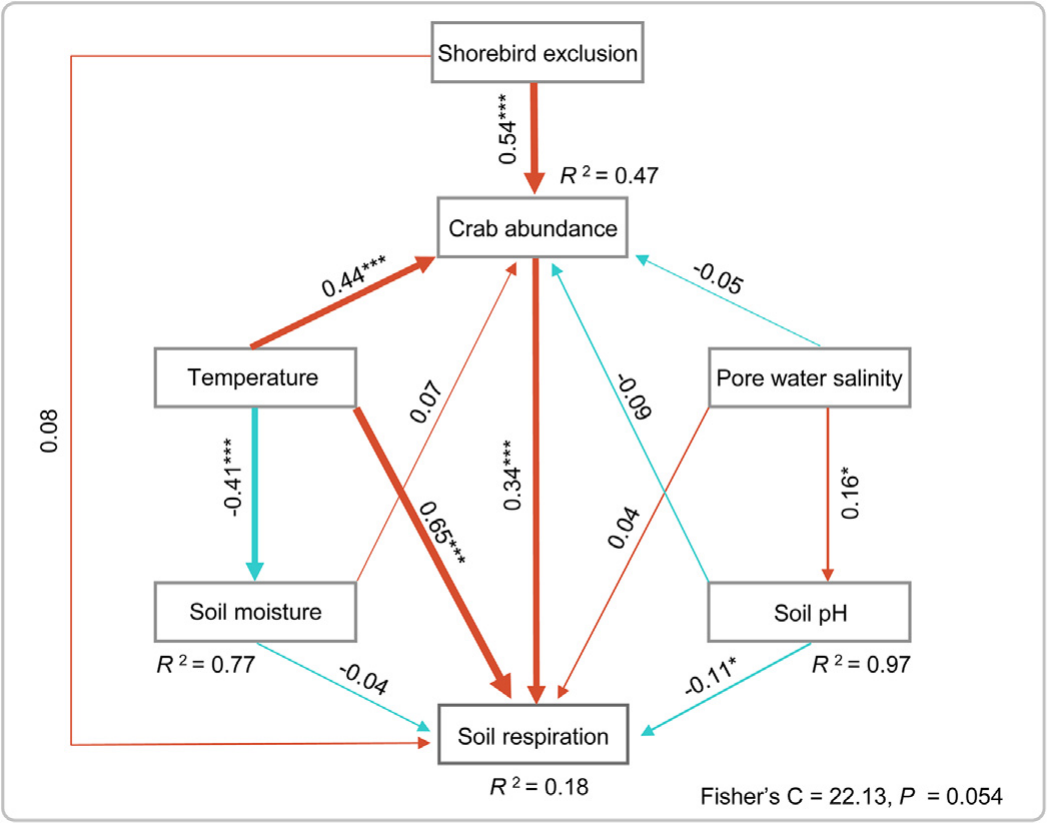
**SEM showing soil respiration as mediated directly and indirectly by shorebirds, crab abundance, and abiotic factors.** Solid red arrows represent positive paths, and blue arrows represent negative paths. Values next to the arrows are the standard correlation coefficients. (*P < 0.05, ^⁎⁎^*P* < 0.01, ^⁎⁎⁎^*P* < 0.001). The marginal *R*^2^ values are reported to represent the proportion of variance explained for each endogenous variable in the model.

In addition to shorebirds, soil respiration was affected by multiple other factors. Univariate regressions showed that soil respiration was positively correlated with crab abundance and soil temperature, negatively correlated with soil moisture and pH, and not significantly correlated with soil pore water salinity (*P* > 0.05, Fig. 2). Consistently, SEM revealed that crab abundance had a significantly positive effect on soil respiration (Fig. 3). Soil temperature had not only a direct positive effect on soil respiration, but also an indirect positive effect on it by increasing crab abundance (Fig. 3). Soil pH had a significantly negative effect on soil respiration, although no significant effects were found for soil moisture and soil pore water salinity (Fig. 3). In addition, the effect size of shorebird exclusion on soil respiration varied with temperature, being greatest when soil temperature was intermediate and smaller when soil temperature was higher or lower (*P* < 0.05, Fig. S1).

Furthermore, our SEM showed that shorebird exclusion did not directly affect soil respiration but significantly increased it by increasing crab abundance (Fig. 3). Although the direct and indirect effects of temperature were strongest in this analysis using our multi-season and multi-year data, the effects of shorebirds remained highly significant and stronger than all other abiotic factors we analyzed (Fig. 3).

### 3.3 Effects of shorebirds on the carbon cycle

**Fig. 4:**
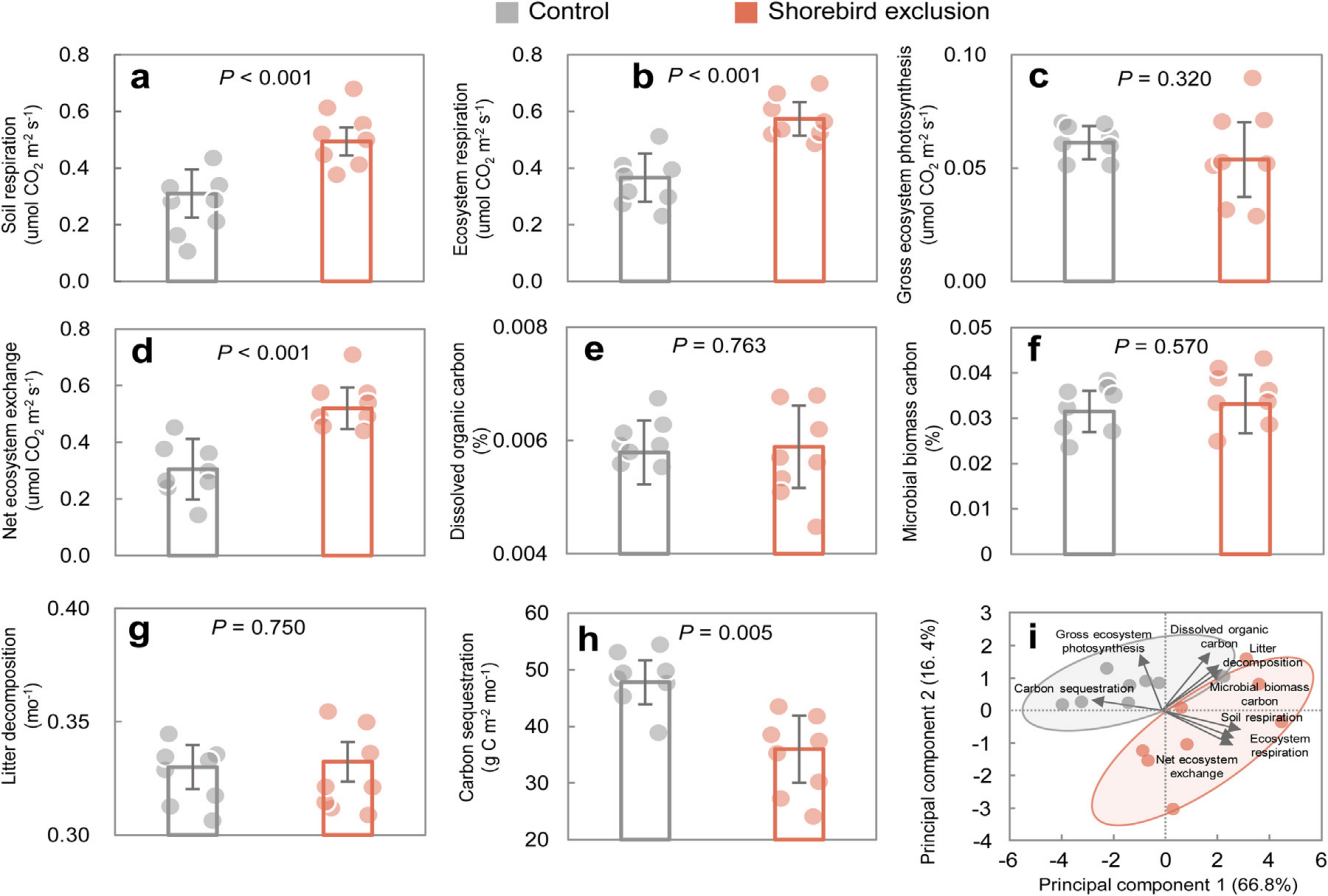
**Effects of shorebird exclusion on the carbon cycle of a coastal wetland.** (a-h) Different metrics of the carbon cycle in control and shorebird exclusion treatments. Data are shown as means ± SE (*n* = 8). *P*-values were calculated using the *t*-test to assess statistical significance between treatments. (i) PCA biplot of the eight carbon cycle metrics in control and shorebird exclusion treatments. PC1 explains 66.8% of the variance and PC2 16.4%. Black arrows represent the loadings of the eight carbon cycle metrics. Ellipses represent the 95% confidence intervals. Colors indicate different treatments.

Shorebird exclusion significantly affected not only soil respiration, but also multiple other processes of the carbon cycle. Compared to control treatments, shorebird exclusion significantly increased soil respiration by 59% (Fig. 4a), ecosystem respiration by 56% (Fig. 4b), and net ecosystem exchange by 70% (Fig. 4d), and significantly decreased soil carbon sequestration by 25% (Fig. 4h). However, it did not affect gross ecosystem photosynthesis (which was minimal in both treatments), soil dissolved organic carbon, soil microbial biomass carbon, and litter decomposition (Fig. 4).

Soil respiration was strongly positively correlated with ecosystem respiration and net ecosystem exchange, and strongly negatively correlated with soil carbon sequestration (Fig. S2), suggesting a predominant role of soil respiration in driving ecosystem respiration, net ecosystem exchange, and soil carbon sequestration. Soil respiration was also significantly positively correlated with soil dissolved organic carbon, microbial biomass carbon, and litter decomposition, although the magnitudes are smaller (Fig. S2). In the PCA on the eight metrics of the carbon cycle, we found that the percentages of explained variance of the first two principal components were 66.8% and 16.4%, respectively (Fig. 4i). Along the first principal component (*df* = 13.85, *P* = 0.004), but not the second (*df* = 13.57, *P* = 0.168), the carbon cycle metrics in shorebird exclusion treatments separated significantly from control treatments.

## 4 Discussion

### 4.1 Shorebird loss can increase wetland CO_2_ emissions

**Fig. 5:**
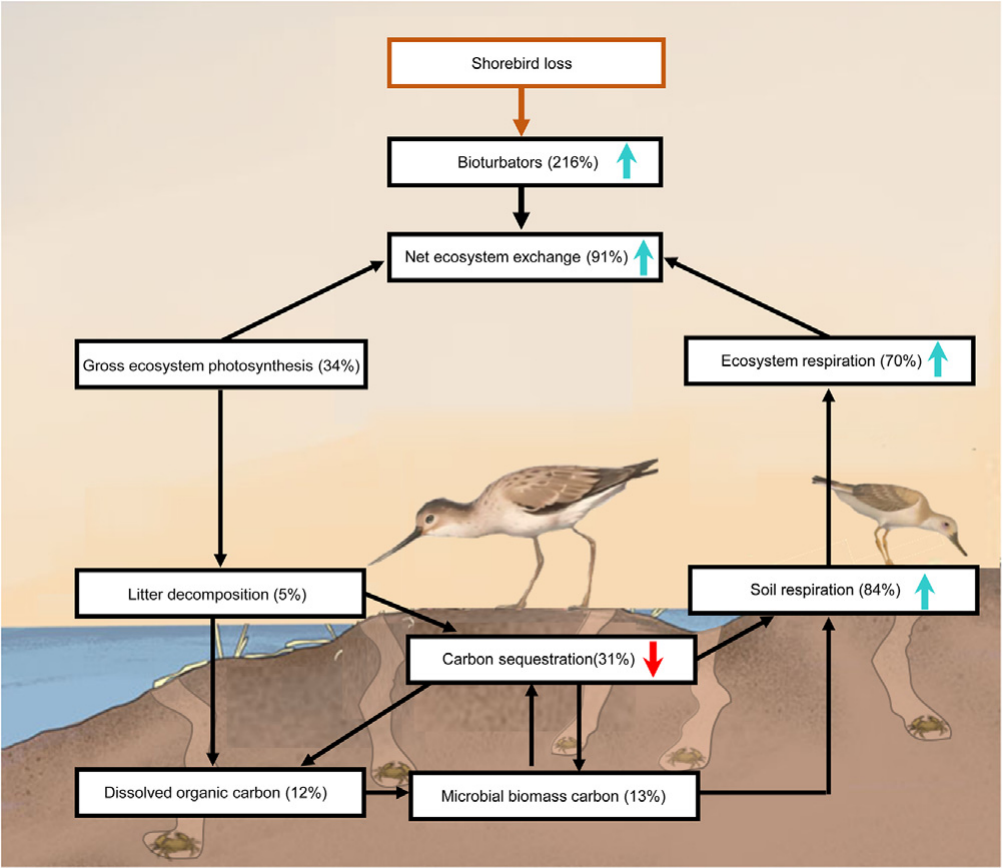
**Effects of shorebird loss on the carbon cycle in the coastal wetland we studied.** Shown are the mean percent differences of the carbon cycle metrics. Significant effects are marked by blue upward arrows (positive effects) or red downward arrows (negative effects) (*P* < 0.05; *P*-values calculated using the *t*-test indicate statistical significance). Effects without arrows are non-significant.

Our study provides one of the first experimental demonstrations that shorebirds can help reduce CO_2_ emissions from coastal wetlands. We found that experimental shorebird exclusion increased soil CO_2_ emissions by 105% on average over four years, an impact that was particularly pronounced in seasons when migratory shorebirds were abundant. We further found that, likely owing to increased soil respiration, shorebird loss significantly increased ecosystem respiration and net ecosystem exchange while reducing soil carbon sequestration (Fig. 5). The profound impacts of shorebirds on carbon dynamics were confirmed in our PCA analysis of all eight carbon cycle metrics, where shorebird exclusion treatments largely diverged from control treatments.

How did shorebirds affect CO_2_ emissions in coastal wetlands? Our study revealed a mechanism that shorebirds reduce wetland CO_2_ emissions by suppressing the abundance of burrowing crabs, and that burrowing crabs can increase soil CO_2_ emissions, as has been demonstrated by previous manipulative studies [29,39]. The observed effects of shorebirds on crab populations can be generally attributed to bird exclusion. First, our previous studies have shown that shorebird exclusion significantly decreased shorebird predation on crabs compared to control treatments [16], suggesting that reduced predation intensity was a key driver of the observed increase in crab abundance in bird exclusion plots. Second, the effect of shorebird exclusion on crab abundance was not constant but increased with increasing shorebird abundance. This effect was strongest during seasons when shorebirds were abundant but nonsignificant when shorebirds were rare at the study site [16]. Third, given the large spaces (10–30 cm) between the nylon strings we used for shorebird exclusion, the shorebird exclusion treatments had a negligible impact on physical factors such as light and water flow, and were thus unlikely to provide crabs with spatial refuges from environmental stressors. This effect of shorebirds on crabs was evident not only for shorebird species that consume crabs (e.g., *Numenius phaeopus, Calidris tenuirostris*, and *Xenus cinereus*), but also for those that do not consume crabs (e.g., *Charadrius alexandrines* and *C. alpina*), owing to the non-consumptive, fear or disturbance effects of shorebirds [16].

Crabs can increase soil CO_2_ emissions through at least three mechanisms. First, crab burrows increase the effective interfacial area between soil and oxygen while also enhancing the effects of electron acceptors (e.g., NO₃⁻, Fe³⁺), thereby promoting organic carbon oxidation [39,52,53]. Second, increasing burrowing activities in crabs may help to promote the survival of aerobic microorganisms by enhancing oxygen supply, thereby increasing soil heterotrophic respiration and accelerating microbial mineralization of organic carbon to CO₂ [53,54,55]. Finally, crab bioturbators can reduce soil carbon burial by destabilizing sediments through their burrowing activities. Additionally, the effects of shorebirds and crabs on soil CO_2_ emissions could be density-dependent. For example, in our study focusing on a coastal wetland without vegetation, soil respiration increased linearly with crab abundance (Fig. 2). Whether this finding applies to other areas where low-density crabs may enhance soil oxygen levels and reduce anoxia stress through active bioturbation, potentially facilitating plant growth and carbon fixation [56], requires further investigation.

Our findings were unlikely to be confounded by the effects of shorebird exclusion on other species (apart from crabs). First, our previous studies showed that shorebird exclusion did not significantly affect other macrofauna (*P* > 0.05) in comparison to control treatments [34]. Second, Spearman’s correlation analysis showed no significant correlation between soil respiration and macrofauna biomass, both of which were quantified in September 2021 (*P* > 0.05, Fig. S3). Furthermore, it is also unlikely that increased soil carbon sequestration in shorebird treatments was due to the input of shorebird droppings. Shorebird droppings were rarely observed at our study site during the multi-year field experiment, although their footprints were frequently observed. These shorebirds primarily use our study site in an intertidal zone as a foraging habitat, while they use more inland areas beyond the high tide zone as roosting habitats where droppings are more frequently observed [57,58].

Our findings underscore the profound impacts of shorebirds on the carbon cycle in coastal wetlands under restoration, where the absence of vegetation led heterotrophic processes (e.g., soil respiration) to dominate the carbon flux. In our study, all planted *Scirpus* were eliminated by crabs in a couple of weeks, leaving no vegetation from 2020 to 2023. Likely owing to the absence of vegetation that fixes carbon dioxide through photosynthesis, shorebird exclusion showed no significant effect on gross ecosystem photosynthesis. Over the past several years, coastal wetlands have become largely unvegetated at our study site due to degradation triggered by droughts, among other factors. In coastal wetlands with existing vegetation, shorebird loss may affect wetland carbon dynamics through additional mechanisms. For example, shorebird loss can increase crab herbivory pressure, thereby suppressing vegetation growth and subsequently reducing the capacity of vegetation to absorb CO₂ through photosynthesis [59]. Furthermore, the negative trophic cascade effect of shorebird loss on vegetation growth can weaken the effectiveness of vegetation in capturing and retaining sediments and associated soil carbon burial [14,60]. The impacts of shorebirds on carbon dynamics in vegetated coastal wetlands, however, have yet to be tested. Future studies examining the relative effects of shorebird loss on carbon dynamics between coastal wetlands with and without vegetation would be particularly insightful.

### 4.2 Shorebird effects relative to abiotic factors

In addition to the strong effects of shorebirds, abiotic factors also play important roles in regulating CO_2_ emissions in coastal wetlands. (i) Our results showed that soil temperature directly and strongly increased soil respiration. Indeed, it is generally accepted that soil respiration increases with increasing soil temperature [45,47,61], as elevated temperatures increase microbial activity and organic matter decomposition [62]. (ii) Our study also found that soil pH directly decreased soil respiration, which corroborates previous studies showing that elevated pH can reduce soil CO₂ emissions by limiting aerobic microbial activity [44,46,63]. (iii) Soil respiration was not affected by soil moisture in our SEM analysis (Fig. 3). This finding is consistent with several manipulative studies showing that soil respiration was not affected by soil moisture [64,65]. In our regression analysis, soil respiration decreased nonlinearly with increasing soil moisture, likely owing to the confounding or compounding effects of covariates. (iv) We found that soil pore water salinity did not significantly affect soil respiration, either. At our study site, high clay contents in sediments may mitigate the effects of soil pore water salinity on soil respiration [66,67].

What is the relative importance of shorebirds versus abiotic factors in mediating soil CO_2_ emissions in coastal wetlands? For their direct effects, we found that soil temperature had a stronger effect on soil respiration than did shorebirds and crab bioturbators. For their indirect effects, both soil temperature and shorebirds can indirectly increase soil respiration by increasing the abundance of crab bioturbators (Fig. 3). Notably, the impact of shorebirds on crab bioturbators was greater than that of soil temperature (∼33%). For their total effects, although temperature played the most important role in regulating soil respiration in coastal wetlands, shorebirds had a significant effect. In other ecosystems, the roles of predators and other consumers in mediating the carbon cycle may also play a profound role, even more important than abiotic factors. For example, it has been shown that predators can significantly reduce CO₂ emissions by 46%−137% through trophic cascades in peatlands [68,69], although comparison with abiotic factors, including temperature, is not available in these studies. In a global synthesis, the effects of herbivores on the carbon cycle in coastal wetland ecosystems have been shown to be comparable with, or even stronger than, changes driven by temperature and other abiotic factors [70].

### 4.3 Enhancing carbon sequestration with trophic rewilding

Our findings have important implications for enhancing the carbon sequestration capacity of degraded coastal wetlands under restoration. We found that experimental exclusion of already depleted shorebird populations strongly decreased carbon sequestration. This effect is driven by a trophic cascade, wherein shorebirds control crab bioturbators that otherwise increase soil CO₂ emissions and destabilize sediments through bioturbation. Top-down effects of shorebirds on soil bioturbators have been demonstrated in multiple other regions. For example, in Canada’s Fundy Bay, semipalmated sandpipers (*Calidris pusilla*) have been shown to reduce densities of mud shrimp (*Corophium volutator*) bioturbators [71]. In Bahia Samborombon on the southwest Atlantic coast, migratory shorebirds, including the American golden-plover (*Pluvialis dominica*), black-bellied plover (*Pluvialis squatarola*), ruddy turnstone (*Arenaria interpres*), and whimbrel (*N. phaeopus*), strongly forage on fiddler crabs (*Uca uruguayensis*) [72]. Furthermore, our previous global synthesis revealed that birds had an overall significant negative effect on the biomass and density of macrofauna in wetlands, a finding evident across different wetland types and macrofauna classes [34]. In these cases, the impacts of shorebirds on bioturbators may also cascade to influence soil carbon sequestration.

Currently, shorebirds are called for conservation and restoration primarily due to their rarity and endangered status [25] and their historical role as passive indicators of wetland health and restoration performance [34,73]. Our findings underscore the profound importance of shorebirds in regulating soil carbon dynamics in coastal wetlands, and provide new, extended incentives for enhancing shorebird conservation and restoration. Our finding suggests that protecting and restoring depleted shorebird populations may be critical to rebuilding trophic structure, reducing CO₂ emissions, and enhancing the capacity of carbon sequestration in degraded coastal wetlands under restoration, including those in the Yellow Sea where shorebird populations have declined by > 80% and up to 8% per year since the mid-1990s [16,25,26].

Our study provides support for the premise of using trophic rewilding to expand NbSs. As a practice of restoring animal populations to recover trophic complexity in ecosystems, rewilding has recently been promoted to achieve self-sustaining ecosystems [74] and has attracted considerable scientific and public interest [75,76]. Ambitious rewilding efforts are already underway in Europe, North America, and remote tropical islands [75,77]. Although there have been previous studies suggesting that maintaining healthy predator populations is essential for protecting carbon stocks [14,77,78], whether trophic rewilding can help mitigate climate change remains controversial, often due to the lack of experimental demonstrations and mechanistic insights. Consequently, the contributions that predators and other wildlife can make remain often unaccounted for in NbSs and the global carbon budget [8]. Our experimental study suggests that rewilding predators of soil bioturbators, which are abundant in many ecosystems such as coastal wetlands, grasslands and forests [29,39,79], could be a valuable approach of NbSs to enhance ecosystem carbon sequestration and help limit climate warming within 2 °C above pre-industrial levels.

## CRediT authorship contribution statement

**Chunming Li:** Writing – review & editing, Writing – original draft, Visualization, Methodology, Investigation, Formal analysis, Data curation, Conceptualization. **Yizhou Sun:** Writing – review & editing, Methodology, Investigation, Data curation. **Peter Müller:** Writing – review & editing. **Mark D. Bertness:** Writing – review & editing. **Baoshan Cui:** Writing – review & editing. **Lijuan Cui:** Writing – review & editing, Writing – original draft, Validation, Supervision. **Bo Li:** Writing – review & editing. **Qiang He:** Writing – review & editing, Writing – original draft, Visualization, Supervision, Project administration, Methodology, Funding acquisition, Conceptualization.

## Declaration of competing interest

The authors declare that they have no conflicts of interest in this work.

## Appendix Supplementary materials

Image, application 1

## References

[1] Donatti C.I., Andrade A., Cohen-Shacham E. (2022). Ensuring that nature-based solutions for climate mitigation address multiple global challenges. One Earth.

[2] Seddon N. (2022). Harnessing the potential of nature-based solutions for mitigating and adapting to climate change. Science.

[3] Mackinnon K., Sobrevila C., Hickey V. (2008).

[4] IUCN, The IUCN Programme 2013–2016, https://iucn.org/sites/default/files/2022-05/wcc-5th-003.pdf.

[5] Griscom B.W., Adams J., Ellis P.W. (2017). Natural climate solutions. Proc. Natl. Acad. Sci. USA.

[6] Girardin C.A., Jenkins S., Seddon N. (2021). Nature-based solutions can help cool the planet-if we act now. Nature.

[7] Miles L. (2021). Nature-based solutions for climate change mitigation. United Nat. Environm. Program. Int. Union. Conserv. Nat..

[8] Schmitz O.J., Sylvén M., Atwood T.B. (2023). Trophic rewilding can expand natural climate solutions. Nat. Clim. Chang..

[9] Jackson R.B., Lajtha K., Crow S.E. (2017). The ecology of soil carbon: Pools, vulnerabilities, and biotic and abiotic controls. Annu. Rev. Ecol. Syst..

[10] Moreno-Mateos D., Barbier E.B., Jones P.C. (2017). Anthropogenic ecosystem disturbance and the recovery debt. Nat. Commun..

[11] Jones H.P., Jones P.C., Barbier E.B. (2018). Restoration and repair of Earth’s damaged ecosystems. Proc. R. Soc. B Biol. Sci..

[12] Shackelford N., Paterno G.B., Winkler D.E. (2021). Drivers of seedling establishment success in dryland restoration efforts. Nat. Ecol. Evol..

[13] Xu C.L., Silliman B.R., Chen J.S. (2023). Herbivory limits success of vegetation restoration globally. Science.

[14] Atwood T.B., Connolly R.M., Ritchie E.G. (2015). Predators help protect carbon stocks in blue carbon ecosystems. Nat. Clim. Chang..

[15] Hammerschlag N., Schmitz O.J., Flecker A.S. (2019). Ecosystem function and services of aquatic predators in the Anthropocene. Trends Ecol. Evol..

[16] Li C.M., Chen J.S., Liao X.L. (2023). Shorebirds-driven trophic cascade helps restore coastal wetland multifunctionality. Nat. Commun..

[17] Jackson J.B., Kirby M.X., Berger W.H. (2001). Historical overfishing and the recent collapse of coastal ecosystems. Science.

[18] Baum J.K., Worm B. (2009). Cascading top-down effects of changing oceanic predator abundances. J. Anim. Ecol..

[19] Bakker E.S., Svenning J.C. (2018). Trophic rewilding: Impact on ecosystems under global change. Phil. Trans. R. Soc. B..

[20] Berzaghi F., Cosimano T., Fullenkamp C. (2022). Value wild animals’ carbon services to fill the biodiversity financing gap. Nat. Clim. Chang..

[21] Mcleod E., Chmura G.L., Bouillon S. (2011). A blueprint for blue carbon: Toward an improved understanding of the role of vegetated coastal habitats in sequestering CO_2_. Front. Ecol. Environ..

[22] Duarte C.M., Losada I.J., Hendriks I.E. (2013). The role of coastal plant communities for climate change mitigation and adaptation. Nat. Clim. Chang..

[23] Chen J., Wang D.Q., Li Y.J. (2020). The carbon stock and sequestration rate in tidal flats from coastal China. Glob. Biogeochem. Cycles..

[24] Fan B.X., Li Y.F. (2024). China’s conservation and restoration of coastal wetlands offset much of the reclamation-induced blue carbon losses. Glob. Change Biol..

[25] Larson C. (2015). Hostile shores. Science.

[26] Studds C.E., Kendall B.E., Murray N.J. (2017). Rapid population decline in migratory shorebirds relying on Yellow Sea tidal mudflats as stopover sites. Nat. Commun..

[27] Sutherland W.J., Atkinson P.W., Butchart S.H. (2022). A horizon scan of global biological conservation issues for 2022. Trends Ecol. Evol..

[28] Ren J.L., Chen J.S., Xu C.L. (2021). An invasive species erodes the performance of coastal wetland protected areas. Sci. Adv..

[29] Xiao K., Wilson A.M., Li H.L. (2021). Large CO_2_ release and tidal flushing in salt marsh crab burrows reduce the potential for blue carbon sequestration. Limnol. Oceanogr..

[30] Yang S.L., Ding P.X., Chen S.L. (2001). Changes in progradation rate of the tidal flats at the mouth of the Changjiang (Yangtze) River, China. Geomorphology.

[31] Tang L., Gao Y., Li B. (2016). *Spartina alterniflora* with high tolerance to salt stress changes vegetation pattern by outcompeting native species. Ecosphere.

[32] Qian W.Q., Chen J.S., Zhang Q. (2021). Top-down control of foundation species recovery during coastal wetland restoration. Sci. Total Environ..

[33] Ma Z.J., Wang Y., Gan X.J. (2009). Waterbird population changes in the wetlands at Chongming Dongtan in the Yangtze River estuary, China. Environ. Manage..

[34] Chen J.S., Li C.M., Wu C.L. (2023). Top-down control of macrofauna: Are waterbirds passengers or drivers in wetlands?. Biol. Conserv..

[35] Touhami F., Bazaïri H., Badaoui B. (2017). The impact of wader predation on benthic macrofauna in Merja Zerga lagoon, Morocco: An exclosure experiment. Wader Study.

[36] Johnston A.S., Meade A., Ardö J. (2021). Temperature thresholds of ecosystem respiration at a global scale. Nat. Ecol. Evol..

[37] Nissan A., Alcolombri U., Peleg N. (2023). Global warming accelerates soil heterotrophic respiration. Nat. Commun..

[38] Liang J., Lu C.Y., Yong Y. (2013). Soil respiration in a subtropical mangrove wetland in the Jiulong River Estuary, China. Pedosphere.

[39] Xiao K., Wu Y.C., Pan F. (2024). Widespread crab burrows enhance greenhouse gas emissions from coastal blue carbon ecosystems. Commun. Earth Environ..

[40] Bertness M.D. (1985). Fiddler crab regulation of *Spartina alterniflora* production on a New England salt marsh. Ecology.

[41] Holdredge C., Bertness M.D., Herrmann N.C. (2010). Fiddler crab control of cordgrass primary production in sandy sediments. Mar. Ecol. Prog. Ser..

[42] Pennings S.C., Selig E.R., Houser L.T. (2003). Geographic variation in positive and negative interactions among salt marsh plants. Ecology.

[43] Fairchild T.P., Walter B., Mutter J.J. (2024). Topographic heterogeneity triggers complementary cascades that enhance ecosystem multifunctionality. Ecology.

[44] Harper C.W., Blair J.M., Fay P.A. (2005). Increased rainfall variability and reduced rainfall amount decreases soil CO_2_ flux in a grassland ecosystem. Glob. Change Biol..

[45] C.K.Wang J.Y.Yang, Zhang Q.Z. (2006). Soil respiration in six temperate forests in China. Glob. Change Biol..

[46] Malik A.A., Puissant J., Buckeridge K.M. (2018). Land use driven change in soil pH affects microbial carbon cycling processes. Nat. Commun..

[47] Haaf D., Six J., Doetterl S. (2021). Global patterns of geo-ecological controls on the response of soil respiration to warming. Nat. Clim. Chang..

[48] Nobbs M., Blamires S.J. (2015). Spatiotemporal distribution and abundance of mangrove ecosystem engineers: Burrowing crabs around canopy gaps. Ecosphere.

[49] Hursh A., Ballantyne A., Cooper L. (2017). The sensitivity of soil respiration to soil temperature, moisture, and carbon supply at the global scale. Glob. Change Biol..

[50] Weissman D.S., Tully K.L. (2020). Saltwater intrusion affects nutrient concentrations in soil porewater and surface waters of coastal habitats. Ecosphere.

[51] Rakhsh F., Golchin A., Al Agha A.B. (2020). Mineralization of organic carbon and formation of microbial biomass in soil: Effects of clay content and composition and the mechanisms involved. Soil Biol. Biochem..

[52] Grow A.K., Schutte C.A., Roberts B.J. (2022). Fiddler crab burrowing increases salt marsh greenhouse gas emissions. Biogeochemistry.

[53] Agusto L.E., Qin G., Thibodeau B. (2022). Fiddling with the blue carbon: Fiddler crab burrows enhance CO_2_ and CH_4_ efflux in saltmarsh. Ecol. Indic..

[54] Chen X.X., Wiesmeier M., Sardans J. (2021). Effects of crabs on greenhouse gas emissions, soil nutrients, and stoichiometry in a subtropical estuarine wetland. Biol. Fertil. Soils.

[55] Kristensen E., Valdemarsen T., de Moraes P.C. (2022). Pneumatophores and crab burrows increase CO_2_ and CH_4_ emission from sediments in two Brazilian fringe mangrove forests. Mar. Ecol. Prog. Ser..

[56] Gittman R.K., Keller D.A. (2013). Fiddler crabs facilitate *Spartina alterniflora* growth, mitigating periwinkle overgrazing of marsh habitat. Ecology.

[57] Rogers D.I., Battley P.F., Piersma T. (2006). High-tide habitat choice: Insights from modelling roost selection by shorebirds around a tropical bay. Anim. Behav..

[58] Jourdan C., Fort J., Pinaud D. (2022). Daytime, tidal amplitude and protected areas influence movements and habitat use on mudflats of wintering black-tailed godwits. Estuar. Coast. Shelf Sci..

[59] Alberti J., Cebrian J., Casariego A.M. (2011). Effects of nutrient enrichment and crab herbivory on a SW Atlantic salt marsh productivity. J. Exp. Mar. Biol. Ecol..

[60] Gacia E., Duarte C.M. (2001). Sediment retention by a Mediterranean Posidonia oceanica meadow: The balance between deposition and resuspension. Estuar. Coast. Shelf Sci..

[61] Bond-Lamberty B., Thomson A. (2010). Temperature-associated increases in the global soil respiration record. Nature.

[62] Kuzyakov Y., Gavrichkova O. (2010). Time lag between photosynthesis and carbon dioxide efflux from soil: A review of mechanisms and controls. Glob. Change Biol..

[63] Orchard V.A., Cook F.J. (1983). Relationship between soil respiration and soil moisture. Soil Biol. Biochem..

[64] Davidson E.A., Nepstad D.C., Ishida F.Y. (2008). Effects of an experimental drought and recovery on soil emissions of carbon dioxide, methane, nitrous oxide, and nitric oxide in a moist tropical forest. Glob. Change Biol..

[65] Zhang X., Zhang Y.P., Sha L.Q. (2015). Effects of continuous drought stress on soil respiration in a tropical rainforest in southwest China. Plant Soil.

[66] Setia R., Marschner P., Baldock J. (2011). Salinity effects on carbon mineralization in soils of varying texture. Soil Biol. Biochem..

[67] Yu Y.X., Li X., Zhao C.Y. (2020). Soil salinity changes the temperature sensitivity of soil carbon dioxide and nitrous oxide emissions. Catena.

[68] Atwood T.B., Hammill E., Greig H.S. (2013). Predator-induced reduction of freshwater carbon dioxide emissions. Nat. Geosci..

[69] Wyatt K.H., McCann K.S., Rober A.R. (2021). Trophic interactions regulate peatland carbon cycling. Ecol. Lett..

[70] He Q., Li H.R., Xu C.L. (2020). Consumer regulation of the carbon cycle in coastal wetland ecosystems. Phil. Trans. R. Soc. B..

[71] Daborn G.R., Amos C.L., Brylinsky M. (1993). An ecological cascade effect: Migratory birds affect stability of intertidal sediments. Limnol. Oceanogr..

[72] Iribarne O.O., Martinez M.M. (1999). Predation on the southwestern Atlantic fiddler crab (*Uca uruguayensis*) by migratory shorebirds (*Pluvialis dominica, P. squatarola, Arenaria interpres*, and *Numenius phaeopus*). Estuaries.

[73] Amano T., Székely T., Sandel B. (2018). Successful conservation of global waterbird populations depends on effective governance. Nature.

[74] Perino A., Pereira H.M., Navarro L.M. (2019). Rewilding complex ecosystems. Science.

[75] Lorimer J., Sandom C., Jepson P. (2015). Rewilding: Science, practice, and politics. Annu. Rev. Env. Res..

[76] Corlett R.T. (2016). Restoration, reintroduction, and rewilding in a changing world. Trends Ecol. Evol..

[77] Svenning J.C., Pedersen P.B., Donlan C.J. (2016). Science for a wilder Anthropocene: Synthesis and future directions for trophic rewilding research. Proc. Natl. Acad. Sci. USA.

[78] Wilmers C.C., Estes J.A., Edwards M. (2012). Do trophic cascades affect the storage and flux of atmospheric carbon? An analysis of sea otters and kelp forests. Front. Ecol. Environ..

[79] Beverly D.P., Huenupi E., Gandolfo A. (2024). The forest, the cicadas and the holey fluxes: Periodical cicada impacts on soil respiration depends on tree mycorrhizal type. Ecol. Lett..

