# Supplementary material for: Shorebird loss increases soil CO2 emissions in coastal wetlands under restoration
Source: Fundam Res. 2025 Sep 15;6(3):1641–9. doi: 10.1016/j.fmre.2025.08.015 (PMC13247496; doi:10.1016/j.fmre.2025.08.015)
Supplement: Supplementary file 1 [file mmc1.docx]

**Supplementary Material**

**Shorebird loss** **increases** **soil CO_2_ emissions in coastal wetlands under restoration**

Chunming Li^a^, Yizhou Sun^a^, Peter Müller^b^, Mark D. Bertness^c^, Baoshan Cui^d^, Lijuan Cui^e,*^, Bo Li^a^, Qiang He^a,*^

^a^ *State Key Laboratory of Wetland Conservation and Restoration, National Observation and Research Station for Wetland Ecosystems of the Yangtze Estuary, Ministry of Education Key Laboratory for Biodiversity Science and Ecological Engineering, School of Life Sciences, Fudan University, 2005 Songhu Road, Shanghai* *200438, China*

^b^ *Institute for Landscape Ecology, University of Münster, Heisenbergstraße 2, 48149 Münster, Germany*

^c^ *Department of Ecology, Evolution and Organismal Biology, Brown University, Providence, RI 02912, USA*

^d^ *State Key Laboratory of Wetland Conservation and Restoration, School of Environment, Beijing Normal University, Beijing 100875, China*

^e^ *State Key Laboratory of Wetland Conservation and Restoration, Institute of Wetland Research, Chinese Academy of Forestry, Beijing 100091, China*

*** Corresponding authors. Email addresses: lkyclj@126.com; he_qiang@hotmail.com


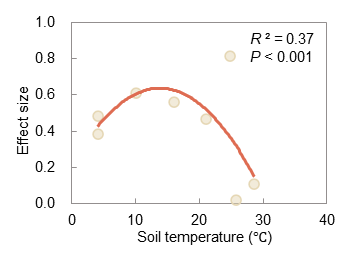


**Fig. S1 The effect size of shorebird exclusion on soil respiration as a function of soil temperature.**

**
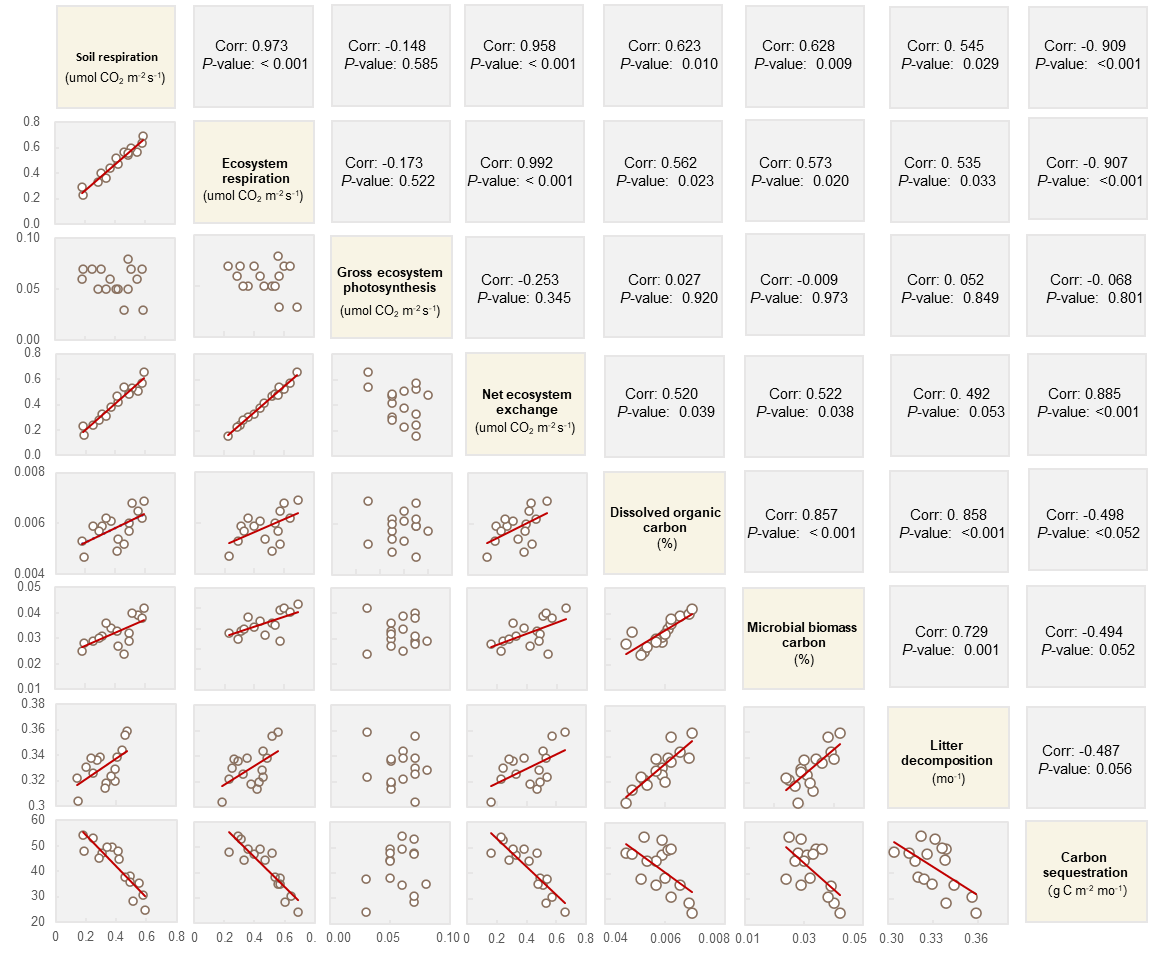
**

**Fig. S2 Correlations among the eight metrics of the carbon cycle.**


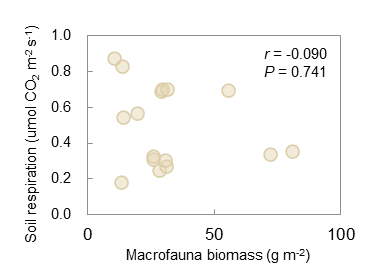


**Fig. S3 Soil respiration was not correlated with** **macrofauna biomass.**

**Table S1 Summary statistics of the linear mixed-effects model** **testing the effects of treatment and season on soil respiration.**

| **Item** | ***df*** | **Chisq** | ***P*-value** |
| --- | --- | --- | --- |
| Intercept | 399.69 | 1 | *P* < 0.0001 |
| Treatment | 10.51 | 1 | *P* = 0.0011 |
| Season | 325.68 | 6 | *P* < 0.0001 |
| Treatment × Season | 23.99 | 6 | *P* = 0.0005 |

**Table S2 Summary statistics of the linear mixed-effects model** **testing the effects of treatment and year on soil respiration.**

| **Item** | ***df*** | **Chisq** | ***P*-value** |
| --- | --- | --- | --- |
| Intercept | 439.01 | 1 | *P* < 0.0001 |
| Treatment | 29.75 | 1 | *P* < 0.0001 |
| Year | 52.31 | 3 | *P* < 0.0001 |
| Treatment × Year | 9.00 | 3 | *P* = 0.0292 |
